# F_O_F_1_-ATPase Motor-Embedded Chromatophore as Drug Delivery System: Extraction, Cargo Loading Ability and Mucus Penetration Ability

**DOI:** 10.3390/pharmaceutics15061681

**Published:** 2023-06-08

**Authors:** Yujing Wu, Bang Lou, Ning Zheng, Xuhui Zhou, Ying Gao, Weiyong Hong, Qingliang Yang, Gensheng Yang

**Affiliations:** 1 College of Pharmaceutical Science, Zhejiang University of Technology, Hangzhou 310014, Chinaloubang96@outlook.com (B.L.);; 2 Zhejiang Moda Biotech Co., Ltd., Hangzhou 310018, China; 3 Department of Pharmacy, Municipal Hospital Affiliated to Taizhou University, Taizhou 318000, China

**Keywords:** F_O_F_1_-ATPase motors, chromatophores, mucus penetration, curcumin

## Abstract

Mucosal drug delivery permits direct and prompt drug absorption, which is capable of reducing undesirable decomposition that occurs before absorption. However, mucus clearance of those mucosal drug delivery systems strongly retards their actual application. Herein, we propose chromatophore nanoparticles embedded with F_O_F_1_-ATPase motors to promote mucus penetration. The F_O_F_1_-ATPase motor-embedded chromatophores were firstly extracted from Thermus thermophilus by using a gradient centrifugation method. Then, the model drug (curcumin) was loaded onto the chromatophores. The drug loading efficiency and entrapment efficiency were optimized by using different loading approaches. The activity, motility, stability and mucus permeation of the drug-loaded chromatophore nanoparticles were thoroughly investigated. Both the in vitro and in vivo studies revealed that the F_O_F_1_-ATPase motor-embedded chromatophore successfully enhanced mucus penetration glioma therapy. This study indicates that the F_O_F_1_-ATPase motor-embedded chromatophore is a promising alternative as a mucosal drug delivery system.

## 1. Introduction

In the past decades, nanoparticulate drug delivery systems (NDDS) have been increasingly applied in pharmaceutical formulation development due to their attractive functionality in delivering actives to the site of action with improved biodistribution and better drug therapy. Although most of these nanoparticulate formulations (NPFs) are administered parenterally, lately more attention has been paid to the design and development of nanoparticulate formulations with better patient compliance. Of these, mucosal cargo delivery based on polymeric nanoparticles has received extensive interest due to its extraordinary potential regarding its drug protection capacity and direct absorption ability. This is especially important in delivering actives with large molecules [1] such as proteins, peptides and vaccines [2]. These mucosal cargo delivery systems could be prepared with versatile formulations and then administered through multiple non-invasive routes such as nasal ones [3], oral ones [4,5], the eye [6], the gastrointestinal tract, airways [7] and the vagina. These routes could be applied both for systemic drug administration and for local treatment of mucosal diseases [8]. However, the mucus layer covered on the mucosa epithelial surface acts as a barrier for the nanoparticle-based delivery system. As a viscoelastic gel, mucus could provide effective protection by trapping foreign particles including pathogens to limit their penetration into the underlying epithelium [9]. Particle-based formulations and drug delivery systems might be trapped in the mucus layer and cleared by the mucus turnover, which is called the mucus clearance. This could tremendously reduce the drug absorption and bioavailability. In the past decade, many efforts have been made to design and develop delivering strategies based on mucoadhesive polymeric materials [9] to avoid potential wash-off and to prolong the residence time of the loaded drugs on the mucus, so as to improve the chance of being absorbed. Those mucoadhesive materials include chitosan [10,11] and its derivative [12], polydopamine [13] and zein [14]. However, there are still ongoing challenges facing those mucoadhesive drug delivery systems that strongly retard their practical applications. First, the applied mucoadhesive materials need to be adequately biocompatible to minimize the side effects that potentially disrupt the mucosa. Most importantly, these polymeric mucoadhesive drug delivery systems cannot provide sufficient propulsion to penetrate the mucus layer and to achieve prompt and complete drug absorption.

Micro/nanomotors (MNMs) [15,16] have the ability to convert energy into propulsion and movement [17], enabling them to be self-propelled vehicles [18]. Thanks to their active mobility and also the miniaturized body size, MNMs have displayed tremendous potential for biomedical applications including biosensing [19], diagnosis, minimally invasive surgery and targeted cargo delivery [20]. However, there are still many ongoing challenges and critical issues that need to be addressed. One is the complex and cost-added fabrication process due to the complicated motor structures [21] for an on-demand integration of multiple functions. Additionally, the biocompatibility and biodegradability of the MNMs are also big challenges owing to their complex compositions [22]. 

As a biomolecular MNM, F_O_F_1_-ATPase MNM [23] lately has attracted extensive interest due to its excellent biocompatibility, low toxicity and nanosized dimension. F_O_F_1_-ATPase has the ability of synthesizing and decomposing ATP [24], during which a proton gradient will be produced and a rotation motion of the F_O_F_1_-ATPase MNM be provoked [25,26]. Impressively, F_O_F_1_-ATPase is easily available and commonly distributed in chloroplasts, mitochondria and bacterial chromatophores (CHR) with liposome structure properties, and it is considered as an ideal drug container and delivery system that could protect the loaded drug from enzymatic hydrolysis [27,28]. Herein, we design and develop a novel nanoparticulate drug delivery system based on F_O_F_1_-ATPase motor-embedded chromatophore, with particular emphasis on the extraction, drug loading, biocompatibility and the mucus penetration abilities of the achieved nanoparticles.

## 2. Materials and Methods

### 2.1. Materials

*Thermus thermophilus*, *E. coli* extracts and artificial mucus were obtained from Moda Biotechnology (Hangzhou, China). Curcumin (CUR), sucrose and acetone were purchased from Sinopharm Chemical Reagent Co., Ltd. (Shanghai, China). C6 cells and Human Nasal Epithelial Cells (HNEpC) were purchased from GuangZhou Jennio Biotech Co., Ltd. (Guangzhou, China). Polycarbonate membrane was purchased from Whatman (Maidstone, UK).

### 2.2. Extraction and Characterization of CHR

#### 2.2.1. Extraction of CHR

*Thermus thermophilus* was incubated in a constant temperature shaker (NR-1112, Shanghai Hainanrong Experimental Equipment Co., Ltd.) at 55 °C and 180 rpm for 20 h and collected by centrifugation. The precipitate of concentrated bacteria was resuspended by an extraction buffer (20 mM Tris-Cl pH 8.0, 100 mM NaCl, 2 mM MgCl_2_, 1 mM DTT) and crushed by an ultrasonic cell crusher (run 5 s, stop 5 s, 450 W) for 60 min. Subsequently, the bacterial fragments were removed by ultracentrifuge (Optima XPN100, Beckman Coulter Inc., Brea, CA, USA) at 23,000 *g* for 45 min. CHRs were separated from the remaining supernatant by being ultracentrifuged at 145,000 g rpm for 45 min. Then, the precipitation was resuspended by the extraction buffer to obtain initial extract chroma for reserve.

Initial extract chroma were purified by density gradient centrifugation. Sucrose solutions of 20%, 40% and 60% were prepared in advance. Each concentration of 2 mL sucrose solutions was added in sequence into the centrifuge tube from 60% to 20%. Additionally, 2 mL of initial extract chroma was added as a top layer. CHRs were separated by being ultracentrifuged at 200,000 *g* for 1 h. Subsequently, the sucrose solutions were recollected for testing ATPase synthesis activity and hydrolytic activity. 

#### 2.2.2. Characterization of CHR

*Size and Zeta potential:* The stoste of CHR was diluted 5 times by a 10 mM phosphate buffer, then the particle size and polymer dispersity index were measured by a particle analyzer (Delsa^TM^ Nano C Particle Analyzer, Beckman Coulter). The Zeta potential of the samples was measured by a laser particle analyzer (Zetasizer Nano ZS ZEN3600, Malvern Panalytical, Malvern, UK). 

*Stability:* The achieved CHR was stored in a cryogenic vial at 4 °C and the particle size and distribution of CHR were followed and monitored for 10 consecutive days to evaluate the stability of the F_O_F_1_-ATPase motors.

### 2.3. Drug Loading

Four different approaches were utilized to load the model drug (curcumin); the details are as follows: 

*Freeze-thaw:* Curcumin-acetone solution (30 mg/mL) was taken and added into CHR suspension at a concentration of 1 mg/mL. After mixing, the solution was transferred to the cryogenic vials and placed in the refrigerator at −80 °C for 30 min. Then, the sample was taken out and placed at room temperature for 30 min to thaw it out. The same procedure was repeated for 3 cycles. Free curcumin was removed by centrifugation.

*Ultrasonication:* Curcumin was firstly prepared into concentrated solution using acetone (30 mg/mL), then incubated with CHR suspension for 30 min. After incubating, the samples were sonicated for 15 min (5 s for work, 5 s for break, 250 W), then incubated for 5 min. The same procedure was repeated for 3 cycles. Free CUR was removed by centrifugation.

*Saponin penetration:* Curcumin-acetone solution (30 mg/mL) was added into CHR suspension at a concentration of 1 mg/mL, and saponin solution (1 mg/mL) was added at a concentration of 50 μg/mL. After mixing, the sample was refrigerated at 4 °C for 1 h. The free curcumin was removed by centrifugation after the completion of permeation.

*Co-incubation:* Curcumin-acetone solution (30 mg/mL) was added into CHR suspension at a concentration of 1 mg/mL and mixed and incubated at room temperature for 48 h. After incubation, free curcumin was removed by centrifugation.

The same procedure as the above three methods was repeated for 3 cycles. The drug loading (DL) and entrapment efficiency (EE) were calculated by recording the absorbance at 420 nm by UV-Vis spectrometer according to the following equations.

$$DL\%=\frac{weight\;of\;drug\;in\;chroma}{weight\;of\;drug\;in\;chroma+weight\;of\;chroma}\times 100\%$$

$$EE\%=\frac{weight\;of\;drug\;in\;chroma}{weight\;of\;free\;drug+weight\;of\;drug\;in\;chroma}\times 100\%$$

### 2.4. Biosafety Evaluations

*Cell viability:* The HNEpC cells were cultured in a minimum essential medium containing 10% Fetal Bovine Serum (FBS) and 1% penicillin-streptomycin at 37 °C with 5% CO_2_ and 95% H_2_O. HNEpC cells were seeded on the 96-well plates at a density of 10,000 cells per well, then were cultured for 24 h and treated with 20× (the highest concentration used in the subsequent experiments) CHR dilutions diluted by PBS. MTT assay was used to assess the cell viability, thus reflecting the safety of CHR.

*Mucosal irritation on SD rats*: Eighteen six-week-old SD-type rats supplied by Zhejiang Academy of Medical Sciences were randomly divided into three groups: (i) the negative control group received 0.9% sodium chloride solution; (ii) the positive control group received 1% sodium deoxycholate; (iii) the experimental group received 20× CHR dilutions. All animals were intranasally administered via bilateral nostrils with 20 μL for each nostril. After consecutive administration, the rats were sacrificed on day 21. The nasal mucosa was removed for observation of bleeding and necrosis, and histopathological observation was performed by hematoxylin-eosin staining. All animal experiments were approved by the laboratory animals ethical committee of the Zhejiang University of Technology (Approval No. MGS20221228062).

*Endotoxin of chroma detecting tests:* Endotoxins liberated by microorganisms are frequent contaminants of protein solutions. To verify the concentration of endotoxin in CHR, a ToxinSensor TM endotoxin detection system was used to detect endotoxin levels of chroma and *E. coli* extracts at the same mass. 

### 2.5. In Vitro Mucus Penetration

#### 2.5.1. In Vitro Motility and Permeation of CHR

The motility and mucosal permeability of the CHR itself was examined using the Franz diffusion cells. The polycarbonate membrane with a 400 nm mesh was immersed in artificial mucus (0.9% NaCl solution containing 5% mucin and 0.01% NaN_3_) [29] and placed between the donor chamber and the receptor chamber to simulate the nasal mucosa. A total of 2 mL of 20× CHR dilution was placed into the donor chamber, and 7.2 mL of PBS was added to the receptor chamber. A control group was set and 2 mL of 20× CHR dilutions was added directly to the receptor chamber and filled with 5.2 mL PBS. A total of 10 μL of solution was taken from the receptor chambers to determine the chemiluminescence intensity with a chemiluminescence detector (Lux-P110, Guangzhou biolight biotech. Co., Ltd., Guangzhou, China), and the ratios of chemiluminescence intensity of each experimental group to the control group at different times were calculated.

#### 2.5.2. In Vitro Permeation of CUR-CHR

A total of 2 mL of CUR-loaded CHR (CUR-CHR) was placed into the donor chamber, and the receptor chamber was filled with PBS containing 0.5% Tween80 and 0.5% sodium dodecyl sulfate. All of the received samples were collected from the receptor chamber and fresh buffer was added until the receptor chamber was filled. The concentration of CUR in the collected samples was determined by HPLC.

### 2.6. In Vitro Efficacy Experiments

CUR-loaded CHRs with different drug concentrations were prepared. The cell lines C6 were cultured in a DMEM low glucose medium containing 10% FBS and 1% penicillin-streptomycin at 37 °C with 5% CO_2_. The C6 cells in the log-growth phase were selected and seeded on the 96-well plates at a density of 10,000 cells per well, which were then cultured for 24 h. When cells grew adherent to above 60% confluence, 200 μL of samples containing different drug concentrations diluted by DMEM without FBS was added (n = 6). After the treatments of 48 h, cell viability was determined by MTT assay and IC_50_ values were obtained. 

### 2.7. In Vivo Antitumor Effect Study

*Animal models:* Twenty six-week-old Sprague Dawley (SD) rats bought from the Zhejiang Academy of Medical Sciences were randomly divided into four groups: blank, model, CUR and CUR- CHR, with five rats in each group. They were housed under normal conditions in the laboratory animals center of the Zhejiang University of Technology, and all procedures were approved by the laboratory animals ethical committee of the Zhejiang University of Technology (Approval No. MGS20221228062). C6 cells were digested with trypsin digestion solution and centrifuged, and the precipitate was resuspended with PBS and centrifuged again to remove residual fetal bovine serum. The washed cell precipitates were resuspended in PBS and the concentration was adjusted to 1 × 10^7^ cells per milliliter. A total of 200 μL of cell suspension was injected subcutaneously into the upper right thigh for each rat. 

The drug was administered on the 7th day after the injection of the C6 cells, as shown in Table 1.

After tumor transplantation, the life status of rats was observed and recorded, including survival, weight change, eating and defecation. At the end of drug administration, one rat in each group was randomly selected to be sacrificed, and the subcutaneous tumor tissue/brain tissue was removed after dissection, cleaned with normal saline and fixed in 4% paraformaldehyde for 24 h to make paraffin sections which were used for hematoxylin-eosin (HE) staining.

### 2.8. Statistical Analysis

The original data were recorded by Microsoft Excel 2010 and analyzed by Origin 2018 and IBM SPSS Statistics 22. Statistical analyses were performed using Student’s *t*-test (*p* < 0.05). All data were expressed as the mean ± standard deviation (SD).

## 3. Results and Discussion

### 3.1. Extraction and Characterization of CHRs with F_o_F_1_-ATPase Motors

**Figure 1:**
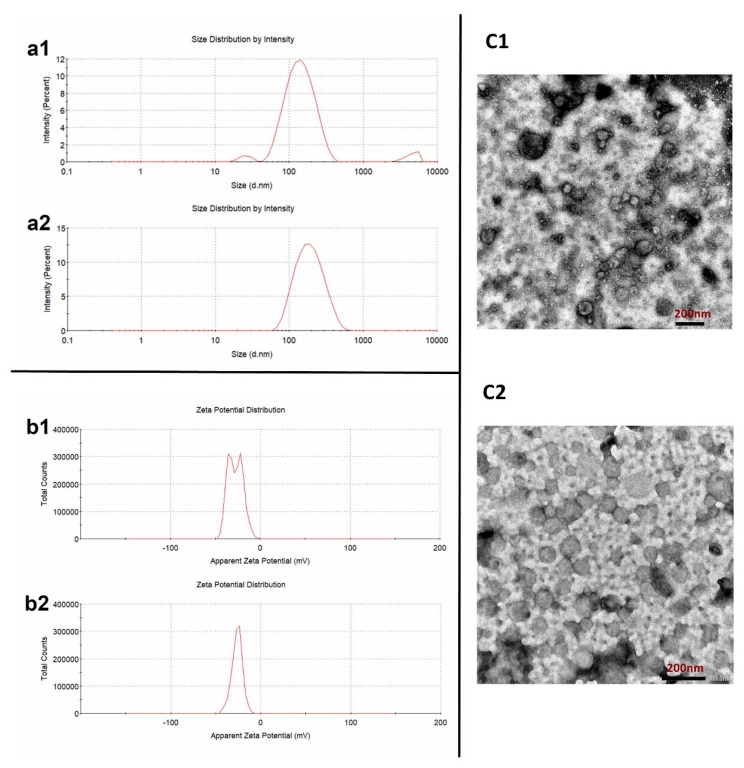
Characterizations of the CHR. a: Particle size of the CHR before (**a1**) and after (**a2**) purification; b: Zeta potential of the CHR before (**b1**) and after (**b2**) purification; c: TEM morphologies of the CHR before (**c1**) and after (**c2**) purification.

As a biomolecular motor, one of the enormous advantages of the F_o_F_1_-ATPase motor is that it can be easily obtained from nature instead of being produced with complicated preparation procedures. As shown in Figure 1, CHRs were successfully extracted from Thermus thermophilus with an average particle size of 129.8 ± 16.5 nm (PDI: 0.285 ± 0.098) and a Zeta potential of −20.4 ± 1.5 mV before purification (Figure 1a1,b1). Moreover, the TEM result (Figure 1c1) showed that the morphology of the initially obtained CHRs was rough and nonuniform, indicating that there might be some impurities. By contrast, after being purified by the gradient purification method, the average particle size of the achieved CHRs was 112.3 ± 2.8 nm with PDI of 0.209 ± 0.010, which was more uniform. This could be confirmed by the TEM result (Figure 1c2), in which most of the particles were spherical with a consistent particle size. The Zeta potential of the purified CHRs also became more uniform, which was −29.3 ± 0.7 mV.

### 3.2. Characterization of the Motility of Drug-Loaded CHR with F_o_F_1_-ATPase Motors

**Figure 2:**
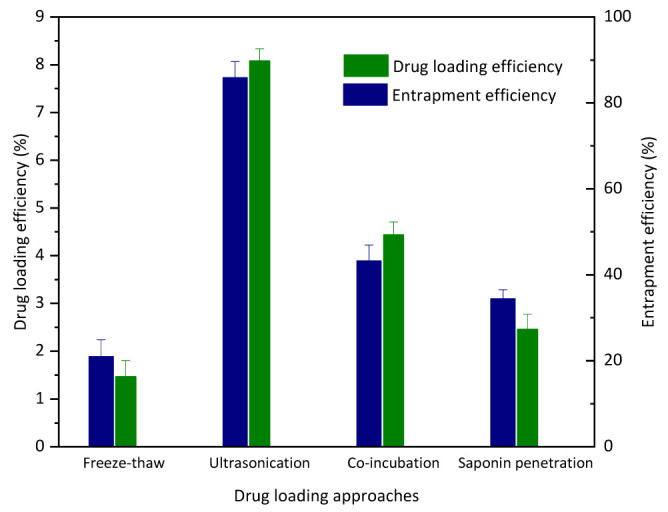
Drug loading capacity (loading efficiency and entrapment efficiency) of CHR with different loading approaches (n = 6).

As a potential cargo delivery system, one prerequisite is the cargo loading capacity. The CHRs extracted from Thermus thermophilus have a liposome structure, which could be an ideal drug container both for hydrophilic actives and for hydrophobic molecules. In order to evaluate the drug loading capacity of the purified CHRs, four different approaches (freeze-thaw, ultrasonication, co-incubation, saponin penetration) were utilized to load the model drug (curcumin). As shown in Figure 2, both the loading efficiency and the entrapment efficiency of CHRs varied greatly. The loading efficiencies of curcumin onto the CHRs with these four approaches were 1.46%, 8.08%, 4.44% and 2.46%, respectively, while the entrapment efficiencies were 20.98%, 85.9%, 43.27% and 34.49%, respectively. These results suggest that the ultrasonication method surpassed others in loading curcumin into the CHRs. This could be explained by the membrane movement of the applied CHR caused by the energy of the ultrasonication, which could entrap drugs more efficiently.

**Figure 3:**
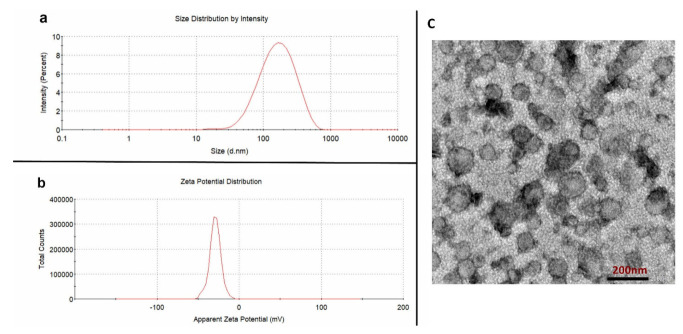
Characterizations of the curcumin-loaded CHR (CUR-CHR). (**a**): Particle size of CUR-CHR; (**b**): Zeta potential of CUR-CHR; (**c**): TEM morphologies of the CUR-CHR.

The particle size and distribution, Zeta potential and morphologies of the curcumin-loaded CHR were also characterized. As shown in Figure 3, the average particle size of the achieved CUR-CHRs was 125.23 ± 3.25 nm (Figure 3a), which was slightly larger than the purified CHRs due to the drug loading. Moreover, the PDI of CUR-CHRs was 0.208 ± 0.008, which was lower than that of CHRs both before and after being purified, indicating that the achieved CUR-CHR particles was adequately uniform. Moreover, the Zeta potential of the CUR-CHRs was more consistent with an average value of −25.65 ± 0.296 mV (Figure 3b). The TEM result (Figure 3c) showed the morphologies of the CUR-CHR particles, most of which were also spherical with a consistent particle size, suggesting that the drug loading process did not change the size and morphology of the CHRs. 

**Figure 4:**
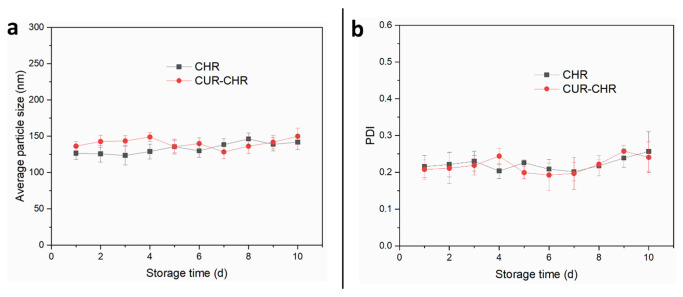
Stability of the drug-loaded CHR. (**a**): Average particle size of the CUR-CHR and CHR after being storage within different time intervals; (**b**): PDI of the CUR-CHR and CHR after being in storage for different time intervals.

Additionally, the stability of CHRs and curcumin-loaded CHRs was also evaluated by monitoring their particle size and distribution over different storage time intervals. As shown in Figure 4, both the particle size (Figure 4a) and PDI (Figure 4b) of the CHRs and CUR-CHRs were sufficiently consistent within 10 days, indicating an adequate stability of both the purified CHRs and drug-loaded CHRs.

**Figure 5:**
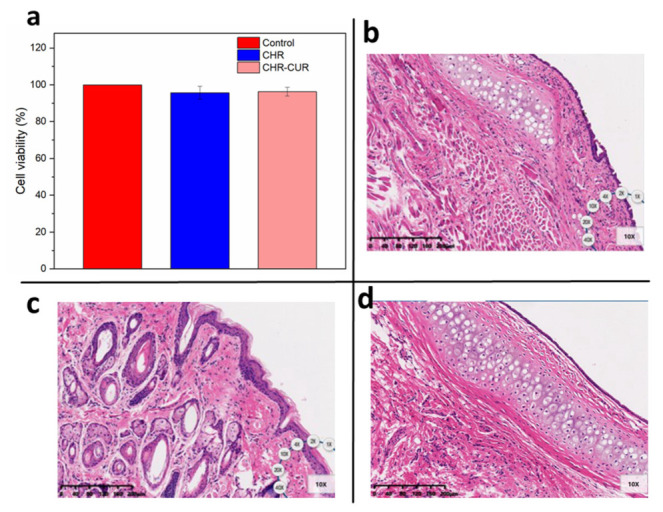
Biocompatibility of free CHRs and drug-loaded CHRs. (**a**) Viability of HNEpC cells after treatments with CHR, CUR-CHR for 24 h (n = 6). (**b**) Tissue morphology of the nasal mucosa from SD rats after dropping saline (negative control), (**c**) 1% sodium deoxycholate (positive control) and (**d**) CHR into the nasal cavity once a day for 21 consecutive days (scale bar: 200 μm).

Biocompatibility is another critical prerequisite for a drug carrier, particularly for the mucosal drug delivery systems. One of the key limitations of the artificial micro/nanomotors is that they have to involve many polymeric materials, which could potentially cause mucosal irritation when being administered through mucosa. On the other hand, F_O_F_1_-ATPase-embedded CHRs are distributed in nature sources such as chloroplasts, mitochondria and bacterial chromatophores, which normally exhibit good biocompatibility. As shown in Figure 5a, different treatments (CHR, CUR-CHR and the control group) did not cause significant difference in the viability of HNEpC cells, indicating that both free CHRs and curcumin-loaded CHRs are sufficiently safe for HNEpC cells. According to the HE staining results (Figure 5b–d), there was no significant damage to the nasal mucosa in the CHR group (Figure 5d) after being treated continuously for 21 days, suggesting an excellent biosafety of CHR. 

**Figure 6:**
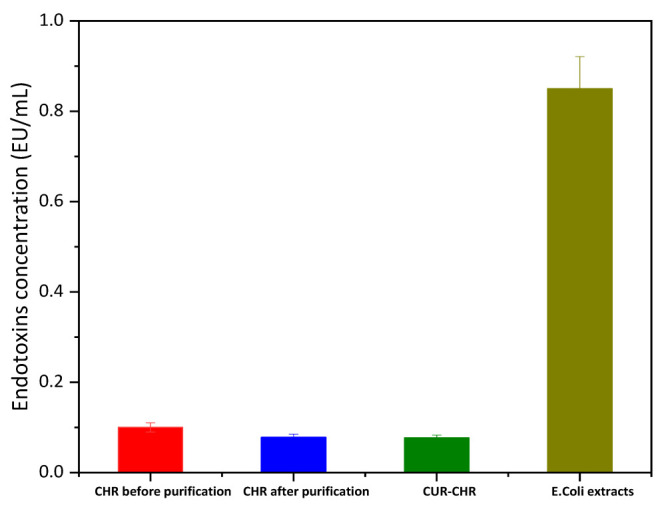
Endotoxin concentration of the CUR-CHR, free CHR before and after purification with E. Coli extracts as control group (n = 6).

Additionally, the endotoxin concentration of both the CUR-CHRs and CHRs before and after being purified was also investigated for the reason that these CHRs were extracted from the bacteria of Thermus thermophilus. As shown in Figure 6, compared to the E. Coli extracts, all the groups of CUR-CHRs and CHR before and after being purified showed a significantly lower endotoxin concentration, suggesting that CHR was a very safe drug carrier that could be administered via many routes, even including intravenous injection. The results also indicated that the purification of the CHRs could further reduce the amount of the endotoxin, while the drug loading process did not cause the endotoxin increase.

### 3.3. Motility of F_o_F_1_-ATPase Motors and Mucus Permeation of Drug-Loaded CHR

**Figure 7:**
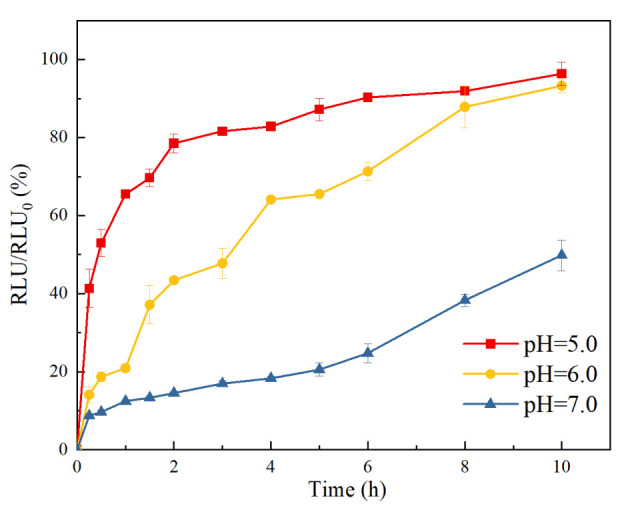
Motility of the CHR samples at different proton concentrations (ratio of the chemiluminescence intensity of the CHR samples in receptor chamber to control group at different pH values within 10 h) (n = 3).

As a proton pump, an F_o_F_1_-ATPase motor could rotate at a high speed driven by a proton gradient, thus propelling the movement of the corresponding CHR [30,31]. As shown in Figure 7, motility of the CHRs, which is the permeation ratio of the CHR samples in the receptor chamber to control group at different pH values, was significantly improved with the increase in proton concentration (with the decrease in pH value). This indicates that F_O_F_1_-ATPase motors possess stronger propulsion and motility within an acid artificial mucus at a lower pH value, which is consistent with our previous study [23]. Additionally, the motion behavior of CHR could be clearly seen in the Supplementary Materials.

**Figure 8:**
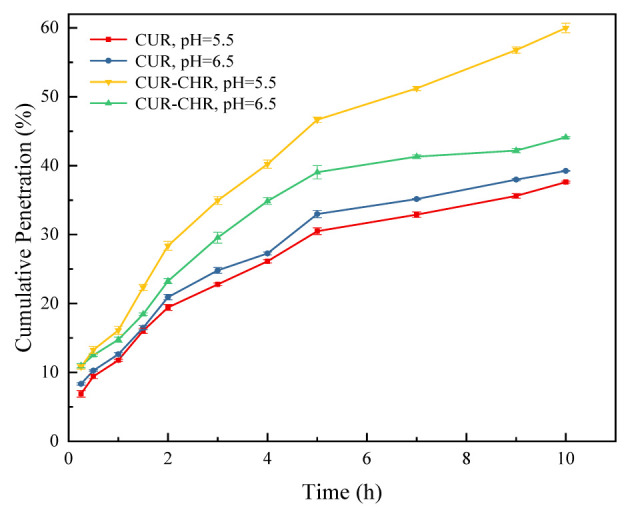
Cumulative drug (CUR) permeation through nasal mucus within 10 h at different pH values with and without CHRs (n = 3).

Additionally, the permeability of drug-loaded CHRs through the artificial mucus was also investigated. As shown in Figure 8, the cumulative permeation of the drug-loaded CHR (CUR-CHR) was much higher compared with that of the free drug (CUR), and a lower pH value resulted in a higher cumulative permeation of the drug-loaded CHR (CUR-CHR), indicating that the presence of the CHR dramatically facilitated the permeation of the drug through the artificial nasal mucus and that a lower pH value allowed a larger enhancement due to the stronger propulsion of the CHR in an acid medium.

### 3.4. Inhibition Effect of Drug-Loaded CHR on C6 Cells

**Figure 9:**
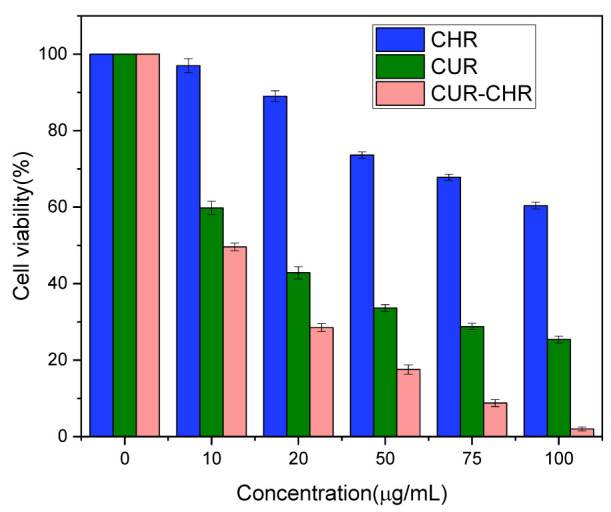
Cell viability of C6 cells after treatments with different concentration of CHR, CUR and CUR-CHR for 48 h (n = 6).

**Figure 10:**
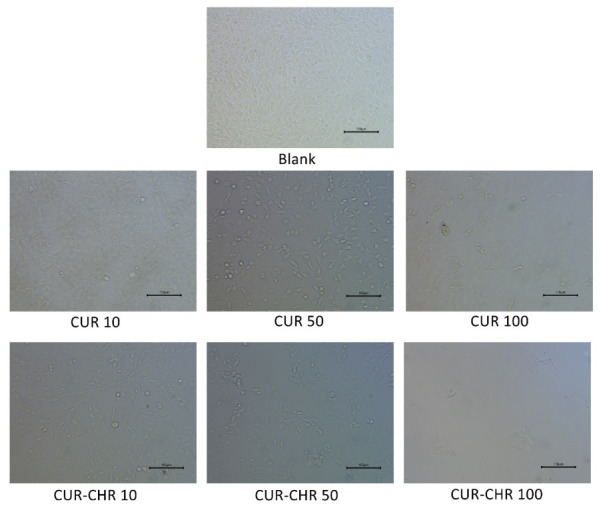
Cell viability and morphologies of C6 cells after treatments with different concentration of CUR and CUR-CHR.

The inhibition effects of free drugs (CUR) and drug-loaded CHR (CUR-CHR) on glioma were examined by MTT assay. As shown in Figure 9, when increasing the concentration of CUR from 0 to 50 μg/mL, the viability of C6 cells dropped below 40%, indicating that curcumin has a strong inhibition effect on the C6 cells. However, further increasing the concentration of CUR did not result in superior inhibition. On the contrary, the CUR-CHR group showed a stronger inhibition effect over the CUR group, particularly in the higher concentrations. This might be caused by the promotion in the cellular uptake of CUR by CHR due to its motor motility. These results could be confirmed by the cell images’ characterization in Figure 10, from which the numbers and morphologies of the cells before and after treatments can be clearly seen.

### 3.5. In Vivo Studies 

**Figure 11:**
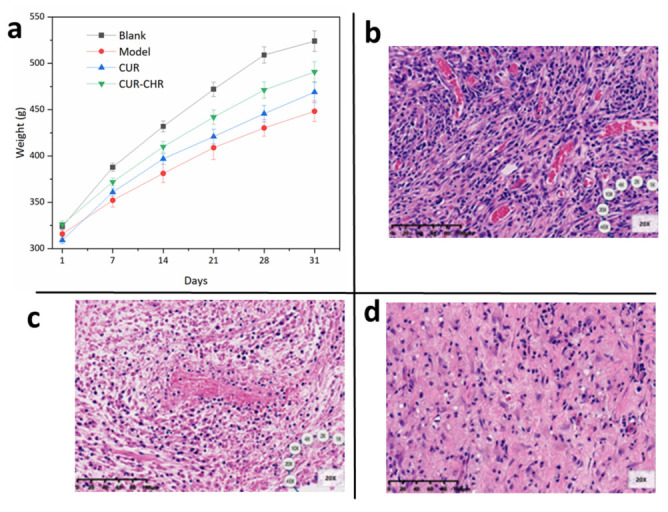
Anti-glioma effect of CUR-CHR. (**a**) Weight change of rats within 30 days of modeling and administration (n = 5); treatments were started on day 7 after molding. (**b**) Tissue morphology of the glioma. (**c**) Tissue morphology of the glioma after treatments with free CUR. (**d**) Tissue morphology of the glioma after treatments with CUR-CHR. (scale bar: 50 μm).

The successful treatments of in vitro tests do not always lead to successful in vivo tests. In order to confirm the anti-tumor ability of this novel drug delivery system, the in vivo therapeutic effects of drug-loaded CHRs embedded with F_O_F_1_-ATPase motors were investigated in a tumor-bearing SD rat model. As shown in Figure 11a, the weight of all the applied rats increased from the beginning to the end of the drug administration, and the CUR-CHR was closest with the blank group, indicating an efficient anti-tumor effect. After being sacrificed, the subcutaneous tumor tissue of rats was removed for paraffin sections and analyzed by HE staining. As can be seen in Figure 11b, subcutaneous glioma tissue cells of the model group grew vigorously and were characterized by large nuclei, nuclear malformation and an abnormal nucleocytoplasmic ratio, indicating that the model was successfully built. After the treatment of free CUR (Figure 11c), tumor cell lysis and necrosis could be seen. This was more obvious in the group of the CUR-CHR (Figure 11d), suggesting that the therapeutic effect of CUR-CHR with the presence of F_O_F_1_-ATPase motor-embedded CHR was better than the free CUR. This could be explained by the motility of the F_O_F_1_-ATPase motors driven by the proton gradient in the microenvironment of the tumors [19]. Compared with the previous in vivo study [23] on the tumor-bearing rat model with drug administered by injection, the intranasally administered drug delivery system proposed by the present study showed a similar or even better anti-tumor efficacy. Practically, the sufficient therapeutic effect of CUR-CHR on the tumor-bearing SD rat model revealed that drug-loaded CHR with intranasal administration is a promising alternative for the treatment of many other solid tumors, such as melanoma.

## 4. Conclusions

In summary, we developed a novel chromatophore nanoparticle embedded with F_O_F_1_-ATPase motors to promote mucus penetration. The F_O_F_1_-ATPase motor-embedded chromatophores were successfully extracted from Thermus thermophilus, and, by using the gradient centrifugation method, the purification improved the qualities of the received CHRs both in terms of particle size uniformity and surface morphologies. Of the four drug loading approaches that were applied, the ultrasonication provided the highest drug loading capacity and entrapment efficiency, and the drug-loaded CHR particles exhibited a sufficient stability, excellent biocompatibility and strong mucus penetration ability. Both the in vitro and in vivo studies revealed that the F_O_F_1_-ATPase motor-embedded chromatophore successfully enhanced C6 cell inhibition and glioma therapy. This study indicates that the F_O_F_1_-ATPase motors-embedded chromatophore is a promising alternative as a mucosal drug delivery system.

## Figures and Tables

**Table 1:** Drug administrations in different groups.

Groups	Drug	Dose/Day	Routes
1 (blank)	/	/	/
2 (model)	/	/	/
3	CUR	6.8 μg	IN
4	CUR-CHR	6.8 μg	IN

## Data Availability

All data generated within this research were included in the manuscript.

## Supplementary Materials

The following supporting information can be downloaded at: https://www.mdpi.com/article/10.3390/pharmaceutics15061681/s1.
